# Enhancing lysosomal biogenesis and autophagic flux by activating the transcription factor EB protects against cadmium-induced neurotoxicity

**DOI:** 10.1038/srep43466

**Published:** 2017-02-27

**Authors:** Huifeng Pi, Min Li, Li Tian, Zhiqi Yang, Zhengping Yu, Zhou Zhou

**Affiliations:** 1 Department of Occupational Health, Third Military Medical University, Chongqing 400038, People’s Republic of China; 2 Brain Research Center, Third Military Medical University, Chongqing 400038, People’s Republic of China; 3 Department of Neurology, Army General Hospital in Lanzhou, Lanzhou 730000, People’s Republic of China; 4 Department of Occupational and Environmental Health, School of Medicine, Guangxi University, Nanning 530004, People’s Republic of China

## Abstract

Cadmium (Cd), a highly ubiquitous heavy metal, is a well-known inducer of neurotoxicity. However, the mechanism underlying cadmium-induced neurotoxicity remains unclear. In this study, we found that Cd inhibits autophagosome-lysosome fusion and impairs lysosomal function by reducing the levels of lysosomal-associated membrane proteins, inhibiting lysosomal proteolysis and altering lysosomal pH, contributing to defects in autophagic clearance and subsequently leading to nerve cell death. In addition, Cd decreases transcription factor EB (TFEB) expression at both the mRNA and protein levels. Furthermore, Cd induces the nuclear translocation of TFEB and TFEB target-gene expression, associated with compromised lysosomal function or a compensatory effect after the impairment of the autophagic flux. Notably, restoration of the levels of lysosomal-associated membrane protein, lysosomal proteolysis, lysosomal pH and autophagic flux through *Tfeb* overexpression protects against Cd-induced neurotoxicity, and this protective effect is incompletely dependent on TFEB nuclear translocation. Moreover, gene transfer of the master autophagy regulator TFEB results in the clearance of toxic proteins and the correction of Cd-induced neurotoxicity *in vivo*. Our study is the first to demonstrate that Cd disrupts lysosomal function and autophagic flux and manipulation of TFEB signalling may be a therapeutic approach for antagonizing Cd-induced neurotoxicity.

Cadmium (Cd) is a widespread non-biodegradable industrial and environmental pollutant that can be greatly concentrated in the food chain^1^. Given its long biological half-life (10–30 years), Cd accumulation in the organism can increase the risk of toxicity. The nervous system is one of the main target organs of Cd toxicity^2^. Cd crosses through the blood-brain barrier (BBB) to reach and accumulate in the brain, producing neurological changes in humans and animals that lead to attention deficits, memory disorders, headache, vertigo, parkinsonism symptoms, and other problems^3^.

Autophagy is an evolutionarily conserved membrane process that results in the transportation of cellular contents to lysosomes for degradation. Autophagic degradation is an important regulator of cellular homeostasis because it mediates the turnover of defective organelles, misfolded or aggregated proteins, and certain long-lived molecules. Perturbations in autophagy during intoxication can arise via at least two mechanisms: **(i)** a blockage in the fusion of autophagosomes with lysosomes; and **(ii)** an impairment of lysosome function that results in the perturbation of basal autophagy^4^. Recently, accumulating evidence has suggested that impaired autophagy plays a vital role in Cd-induced neurotoxicity^5^,^6^; however, its cell-type specificity and mechanism of induction remain unclear.

The basic helix-loop-helix leucine zipper transcription factor TFEB (transcription factor EB) has emerged as a master gene that regulates the number and function of lysosomes and autophagy^7^. TFEB controls the transcription of target genes that are closely related to lysosomal structure and function, including hydrolases, lysosomal membrane proteins and the V-ATPase complex^8^. Furthermore, because TFEB promotes autophagosomal–lysosomal fusion, this transcription factor has the potential to resolve autophagic build-up and maintain the degradation pathway. Notably, *in vivo* TFEB overexpression also increased autophagic flux and lysosomal function in neurodegenerative disorders^9^,^10^,^11^,^12^.

In this study, we characterized the effect of TFEB overexpression on lysosomal and autophagic accumulation in Cd-induced neurotoxicity. TFEB expression markedly increased the levels of lysosomal membrane protein, preserved lysosomal protease activity, maintained the lysosomal pH level and restored cell viability. In addition, TFEB overexpression alleviated autophagic pathology by promoting autophagosome-lysosome fusion and the removal of autolysosomes. Thus, the modulation of TFEB activity holds promise for the development of better therapy for this devastating disorder.

## Results

### Cd inhibits autophagic flux in Neuro-2a cells

**Figure 1:**
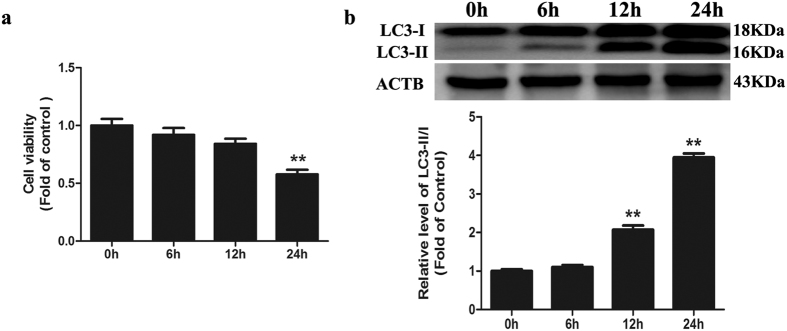
Cd induces autophagosome accumulation in cultured Neuro-2a cells. (**a**) Cell viabilities were determined using a CCK-8 Kit after Neuro-2a cells were treated with 50 μM Cd for different times (0, 6, 12, or 24 h). (**b**) A representative immunoblot and quantification analysis of LC3 as assayed after Neuro-2a cells were treated with 50 μM Cd for different times (0, 6, 12, or 24 h). ACTB was used as an internal standard for protein loading. The results are expressed as a percentage of the control. The values are presented as the means ± SEM. **p < 0.01 versus the control group. (n = 4).

**Figure 2:**
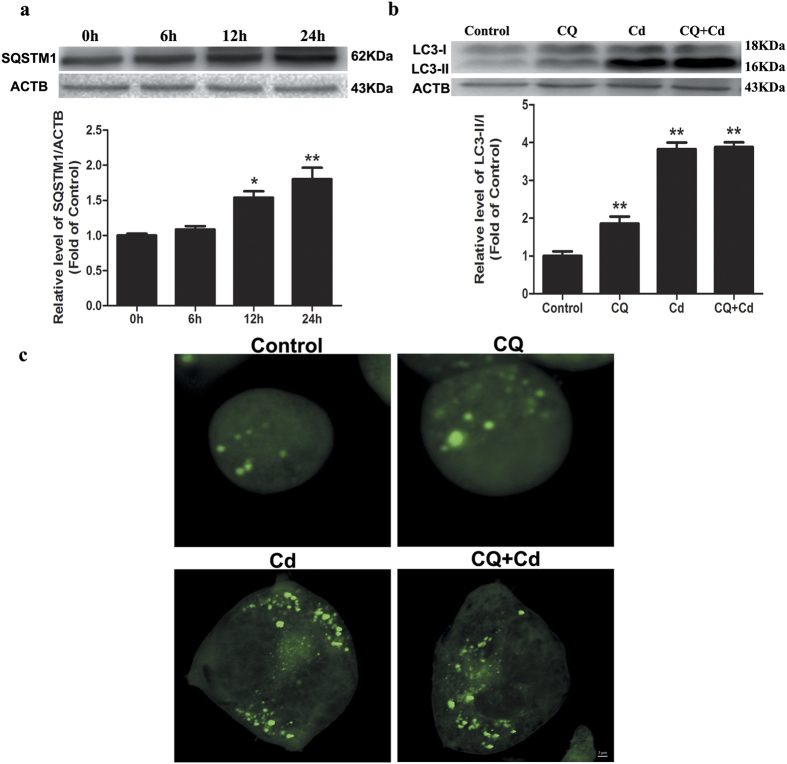
Cd inhibits autophagic flux in cultured Neuro-2a cells. (**a**) A representative immunoblot and quantification analysis of SQSTM1 as assayed after Neuro-2a cells were treated with 50 μM Cd for different times (0, 6, 12, or 24 h). ACTB was used as an internal standard for protein loading. (**b**) A representative immunoblot and quantification analysis of LC3 or (**c**) GFP-LC3 puncta assayed after Neuro-2a cells were treated with Cd (50 μM) in the absence or presence of CQ (50 μM) for 24 h. The results are expressed as fold changes compared with the control. The values are presented as the means ± SEM. *p < 0.05, **p < 0.01 versus the control group. (n = 4).

In this study, we investigated the neurotoxic effect of Cd in Neuro-2a cells and found that Cd induced cell death in a time-dependent manner. The treatment of cells with 50 μM Cd for 6, 12, and 24-h caused approximately 8%, 16%, and 42% reductions in cell viability, respectively (Fig. 1a). Because autophagy has been proposed to play a pivotal role in Cd-mediated neurotoxicity, we determined whether Cd could induce autophagy in Neuro-2a cells. To confirm the progression of autophagy, we directly detected GFP-LC3 distribution. After treatment with 50 μM Cd for 24 h, GFP-LC3 changed from the dispersion state into puncta (Fig. 2c and S3a), suggesting that Cd converted the soluble form of LC3-I to the lipidated LC3-II form associated with autophagosomes. Immunoblotting results also indicated that Cd could up-regulate the expression of LC3-II in a time-dependent manner (Fig. 1b). Both the induction and suppression of autolysosomal maturation resulted in increased numbers of GFP-LC3 puncta or accumulation of LC3-II. To distinguish whether autophagosome accumulation was caused by autophagy induction or by inhibition of downstream steps, we performed an autophagic flux assay^13^. The SQSTM1/p62 protein specifically interacts with LC3 for its degradation by autophagolysosomes. At the cellular level, p62 was negatively correlated with autophagic flux^14^. The increase in the p62 protein level together with the increase in the LC3-II level suggests that autophagic flux is impaired in the Cd-treated group (Fig. 2a). Chloroquine (CQ) prevents autophagy at a late stage by inhibiting fusion between autophagosomes and lysosomes^15^. To further detect autophagic flux, we measured the level of LC3-II or GFP-LC3 puncta in the absence or presence of CQ. We found that 50 μM Cd-induced accumulation of LC3-II or GFP-LC3 puncta was not significantly enhanced in the presence of CQ (Fig. 2b,c and S3a). Taken together, these results suggest that Cd inhibited cellular autophagic processes in Neuro-2a cells.

### Cd blocks autophagosome-lysosome fusion in Neuro-2a cells

**Figure 3:**
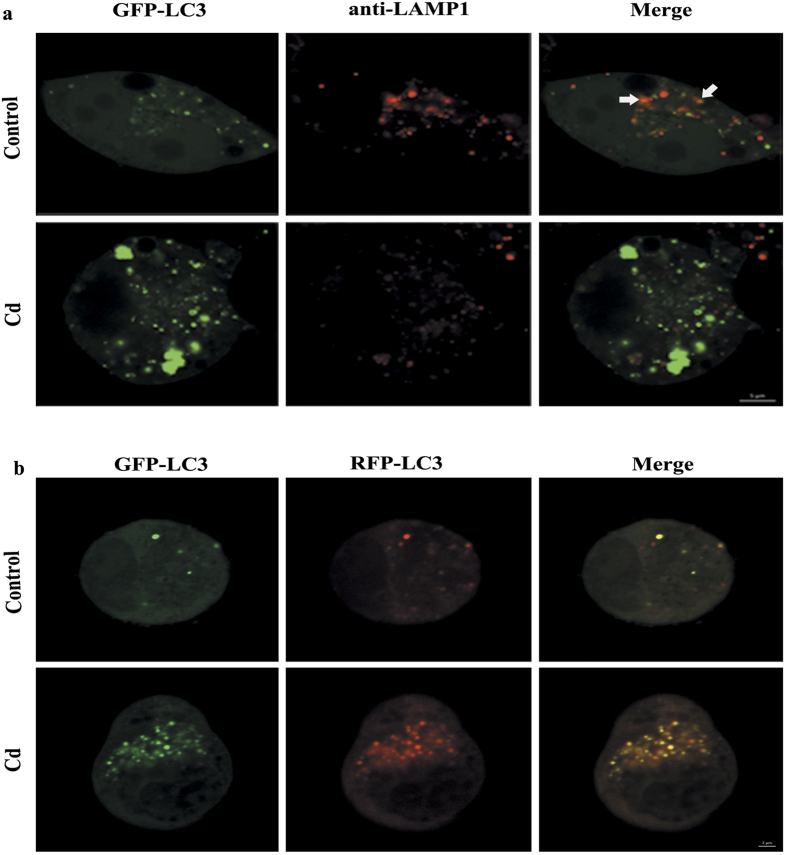
Cd blocks autophagosome-lysosome fusion in cultured Neuro-2a cells. (**a**) Immunofluorescence of Neuro-2a with anti-LAMP1 antibody and colocalization with GFP-LC3B puncta after Cd (0, or 50 μM) treatment for 24 h; (**b**) The colocalization of GFP-LC3 puncta and RFP-LC3 puncta was examined by confocal microscopy after Neuro-2a cells that were infected using a Premo™ Autophagy Tandem Sensor RFP-GFP-LC3B Kit were treated with Cd (50 μM) for 24 h. Arrows indicate colocalization between LC3 and LAMP1. The results are expressed as fold changes compared to the control. The values are presented as the means ± SEM. **p < 0.01 versus the control group. (n = 6).

The fusion of the autophagosomes with lysosomes is an important stage of autophagic flux. To determine whether Cd would affect the fusion of autophagosomes with lysosomes, we performed immunostaining for lysosomal-associated membrane protein 1 (LAMP-1), a lysosomal outer membrane protein, and quantified the colocalization of LAMP-1 with GFP-LC3 puncta after Cd treatment. We found that cells treated with 50 μM Cd decreased the colocalization of GFP-LC3 puncta with LAMP-1 compared with control cells (Fig. 3a and S3b). To further confirm that Cd might impair the maturation of autophagosomes, we transfected Neuro-2a cells with tandem fluorescent GFP-RFP-LC3 (tf-LC3). Thus, LC3-II positive autophagosomes were labeled with both GFP and RFP signals, shown as yellow puncta. After fusion with lysosomes, autolysosomes are shown as red puncta because GFP loses its fluorescence in acidic pH conditions^16^. Consistent with the GFP-LC3 and LAMP-1 colocalization results, Cd treatment led to an obvious increase in the number of yellow (both GFP and RFP fluorescence) dots per cell (Fig. 3b and S3c). Taken together, these results provide strong evidence that Cd blocks autophagosome-lysosome fusion and impairs the clearance of autophagosomes, resulting in their accumulation in the cytosol.

### Cd impairs lysosomal function in Neuro-2a cells

**Figure 4:**
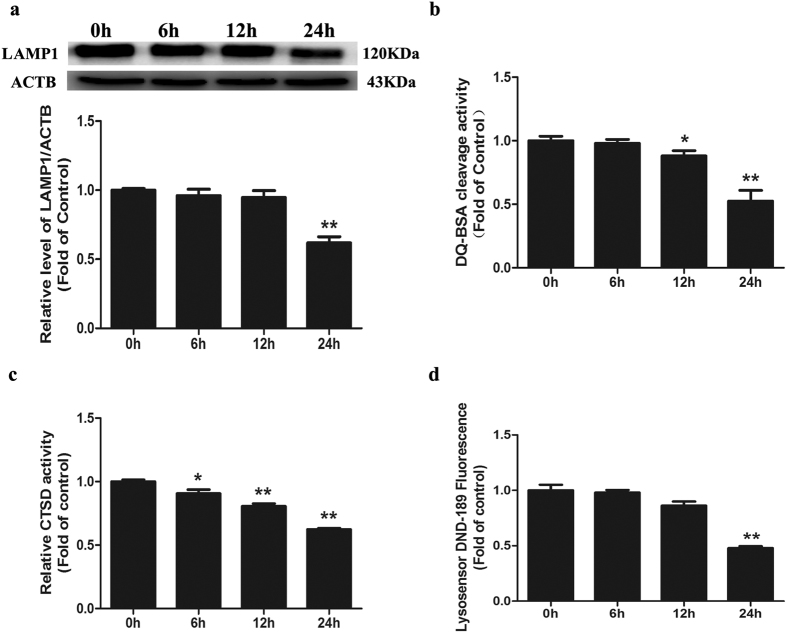
Cd impairs lysosomal function in cultured Neuro-2a cells. (**a**) Representative western blot and quantification analysis for LAMP1 protein in Neuro-2a cells treated with 50 μM Cd for different times (0, 6, 12, or 24 h). ACTB was used as an internal standard for protein loading. (**b**) DQ-BSA cleavage activity of Neuro-2a cells treated with 50 μM Cd for different times (0, 6, 12, or 24 h). (**c**) CTSD activity of Neuro-2a cells treated with 50 μM Cd for different times (0, 6, 12, or 24 h). (**d**) Lysosensor DND-189 fluorescence intensity of Neuro-2a cells treated with 50 μM Cd for different times (0, 6, 12, or 24 h). The results are expressed as fold changes compared with the control. The values are presented as the means ± SEM. *p < 0.05, **p < 0.01 versus the control group. (n = 4).

Lysosomal protein degradation constitutes the final step in both bulk and cargo-specific autophagy^17^. We assessed whether Cd affects the general lysosomal function to inhibit autophagic flux. **(1)** LAMP1, a glycoprotein abundantly expressed on the lysosomal membrane, is a commonly used marker of lysosomal amounts or morphology. Indeed, in Neuro-2a cells treated with 50 μM Cd for 6, 12, and 24 h, LAMP1 decreased significantly (Fig. 4a), suggesting that Cd decreased the amount or size of lysosomes. **(2)** DQ-BSA is delivered to the late endosome/lysosome and is subject to proteolysis by lysosomal enzymes, leading to quantifiable fluorescence. Thus, the fluorescence intensity of DQ-BSA can be used to visualize lysosomal proteolytic activity^18^. We found that Cd caused a noticeable decrease in the fluorescence intensity released from DQ-BSA in a time-dependent manner (Fig. 4b), indicating that intracellular proteolytic activity was inhibited by Cd. **(3)** Cathepsin D (CTSD), a typical cysteine lysosomal protease, plays a critical role in neuronal death through lysosomal leakage or the autophagic pathway in neurons. CTSD activity was reduced in a time-dependent manner with Cd treatment (Fig. 4c). (4) Given that a low pH in the lysosome is required for lysosomal enzyme activity, we applied LysoSensor Green DND-189 to qualitatively measure the lysosomal pH. LysoSensor Green DND-189 permeates cell membranes and accumulates in acidic intracellular organelles, and its fluorescence increases or decreases in acidic or alkaline environments, respectively^19^. As shown in Fig. 4d, compared with the control group, the Cd-treated cells showed a marked decrease in green fluorescence intensity in a time-dependent manner. Based on our data from LysoSensor Green DND-189 staining, Cd increased the pH of acidic compartments. Taken together, these results suggest that Cd impairs lysosomal function by reducing the levels of lysosomal-associated membrane proteins, inhibiting lysosomal proteolysis and altering lysosomal pH in Neuro-2a cells.

### Lysosomal stress mediated by Cd leads to a compensatory lysosomal biogenesis transcriptional response

**Figure 5:**
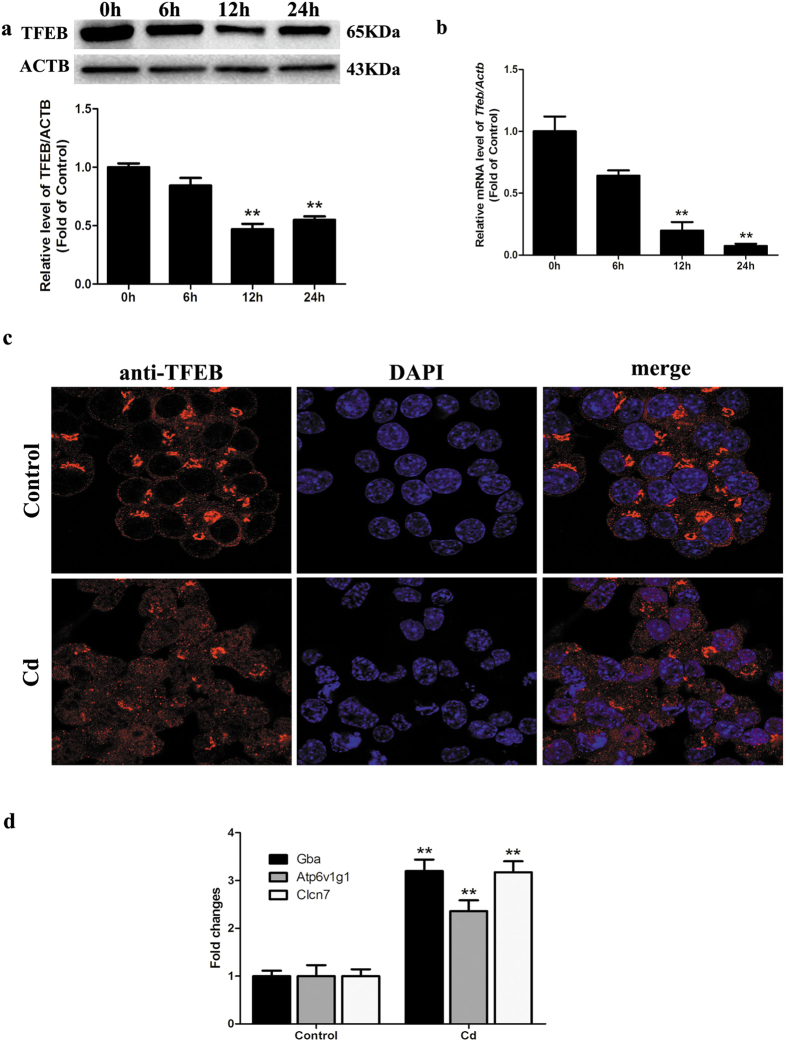
Cd decreases TFEB expression in cultured Neuro-2a cells. (**a**) Representative western blot and quantification analysis for LAMP1 protein in Neuro-2a cells treated with 50 μM Cd for different times (0, 6, 12, or 24 h). ACTB was used as an internal standard for protein loading. (**b**) qRT-PCR analysis revealing fold expression of TFEB in Neuro-2a cells treated with 50 μM Cd for different times (0, 6, 12, 24 h). (**c**) Immunofluorescence of Neuro-2a cells incubated with anti-TFEB antibody and DAPI after Cd (50 μM) treatment for 24 h; scale bar: 20 μm. (**d**) Relative mRNA levels of TFEB target genes were determined after Neuro-2a cells were treated with 50 μM Cd for 24 h. The results are expressed as fold changes compared with the control. The values are presented as the means ± SEM. **p < 0.01 versus the control group. (n = 6).

The recently discovered CLEAR gene network that activates lysosomal biogenesis and autophagic flux coordinated by TFEB prompted us to investigate whether Cd treatment may affect TFEB expression in Neuro-2a cells. As shown in Fig. 5a and b, Cd treatment resulted in a significant decrease in TFEB mRNA and protein levels. Furthermore, the induction of lysosomal stress by potent lysosomal inhibitors, such as chloroquine, initiates a compensatory increase in lysosomal biogenesis via TFEB nuclear translocation^20^,^21^. In light of our data demonstrating the development of lysosomal dysfunction in Cd-induced neurotoxicity, we first evaluated the effects of such stressors on the lysosomal biogenesis transcriptional response. Indeed, such a response appears to be conserved in Neuro-2a cells, as 50 μM Cd treatment for 24 h leads to the induction of a panel of lysosomal TFEB gene targets, namely, GBA, ATP6V1G1 and CLCN1 (Fig. 5d) concomitant with nuclear translocation of TFEB (Fig. 5c and S3d).

### *Tfeb* overexpression attenuates Cd-inhibited lysosomal function and restores autophagic flux in Neuro-2a cells

**Figure 6:**
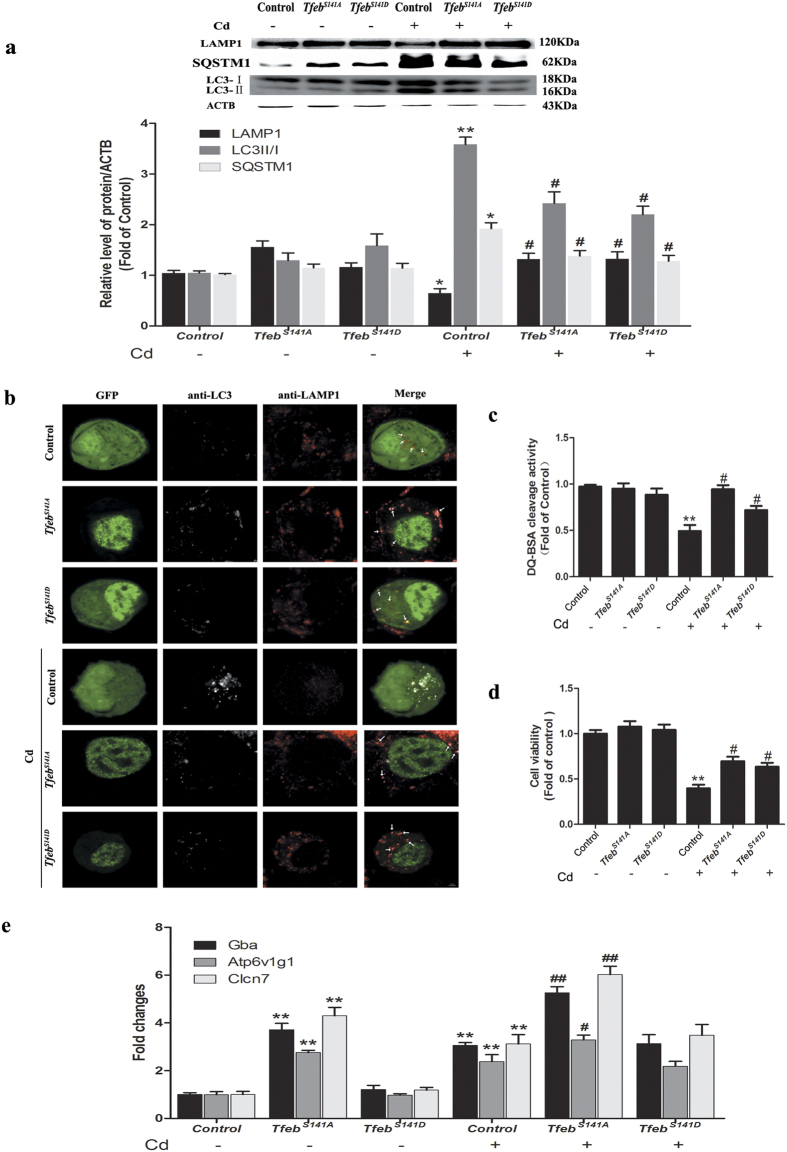
Overexpression of *Tfeb* attenuates Cd-inhibited lysosomal function and restores autophagic flux in cultured Neuro-2a cells. Neuro-2a cells were treated with Cd (50 μM) after transfection with pEGFP-N1-*Tfeb*^*S141A*^, pEGFP-N1-*Tfeb*^*S141D*^ and control plasmids. (**a**) A representative immunoblot and quantification analysis of LAMP1, LC3 and SQSTM1 in Neuro-2a cells. ACTB was used as the internal standard for protein loading. (**b**) Immunofluorescence of Neuro-2a cells with anti-LC3B and anti-LAMP1 antibody. Arrows indicate colocalization between LC3 and LAMP1; scale bar: 2 μm. (**c**) DQ-BSA staining of Neuro-2a cells. (**d**) Cell viability of Neuro-2a cells. (**e**) Relative mRNA levels of TFEB target genes were determined. The results are expressed as fold changes compared with the control. The values are presented as the means ± SEM. *p < 0.05, **p < 0.01 versus the control group, ^#^p < 0.05, ^##^p < 0.01 versus the Cd (50 μM) group. (n = 6).

Given the inability of Neuro-2a cells to mount a robust compensatory transcriptional response to Cd, we next determined whether the restoration of TFEB could rescue Cd-inhibited lysosomal function and autophagic flux. To answer this question, we examined the effect of enforced overexpression of *Tfeb*^*S141A*^, the S141A mutant of TFEB that has recently been shown to cause stronger induction of the autophagic-lysosomal system in Neuro-2a cells compared with WT-*Tfeb* using an inducible strategy^22^,^23^. Here, overexpression of *Tfeb*^*S141A*^ led to a significant increase in the level of LAMP1. Consistently, the degradation of p62 and LC3II was enhanced by *Tfeb*^*S141A*^ overexpression (Fig. 6a). In parallel, *Tfeb*^*S141A*^-overexpressing cells exhibited marked increases in colocalization of LC3B with LAMP1 (Fig. 6b and S3e), demonstrating the rescue of autophagosome-lysosome fusion. Moreover, as assayed by DQ-BSA staining, lysosomal protease activity increased to levels almost comparable to normal levels as a result of *Tfeb*^*S141A*^ overexpression (Fig. 6c). Collectively, these results indicate that *Tfeb* overexpression efficiently protected against Cd-inhibited lysosomal function and autophagic flux.

Based on the above results, *Tfeb* overexpression can be used to restore autophagic flux and lysosomal function. Impaired autophagy is associated with the aggregation of proteins and other cellular constituents, eventually leading to cell death. Therefore, to determine whether autophagy and lysosome dysregulation resulted from Cd-induced neurotoxicity, transfected cells were assessed for Cd-induced cell death. As expected, upon Cd treatment, *Tfeb*^*S141A*^ overexpression markedly increased cell viability compared with Cd-treated cells transfected with a control plasmid (Fig. 6d), indicating that the improvement of the autophagy-lysosome pathway via *Tfeb* overexpression played a pivotal role in rescuing Cd-induced neurotoxicity.

### The protective effect of TFEB is independent, at least in part, of its role as a nuclear transcription factor in Cd-treated Neuro-2a cells

Recently, the transcription of TFEB-regulated genes was shown to depend on its localization to the nucleus^24^. To distinguish whether translocation of TFEB to the nucleus is required to reverse lysosomal function and autophagic flux, we generated two mutant *Tfeb* plasmids. The *Tfeb*^*S141A*^ mutant exhibited significantly increased nuclear localization compared with control plasmids; conversely, the phosphomimetic mutant *Tfeb*^*S141D*^ was unable to translocate into the nucleus (Fig. S1a). To our surprise, compared with the effectiveness of nuclear *Tfeb*^*S141A*^, cytosolic *Tfeb*^*S141D*^ remained effective in reversing lysosomal function and autophagic flux in Cd-treated Neuro-2a cells (Fig. 6a–d and S3e).

### TFEB is a new potential therapeutic target for Cd-induced neurotoxicity *in vivo*

To determine the role of TFEB-dependent lysosomal function and autophagic flux *in vivo*, neuron-specific transgenic expression of *Tfeb* was used to investigate the therapeutic potential of *Tfeb* gene transfer for treating Cd neurotoxicity. For the specific expression of *Tfeb* in neurons, a SYN promoter was used to drive *Tfeb* expression in the adeno-associated virus (AAV) vector [AAV-*Tfeb*^*S141A*^-SYN-EGFP and AAV-*Tfeb*^*S141D*^-SYN-EGFP]. AAV carrying a SYN promoter that drives EGFP expression served as the control. The efficacy of *Tfeb* overexpression was confirmed using immunofluorescence staining of cortical sections with a TFEB antibody. As expected, the *Tfeb*^*S141A*^ mutant translocated into the nucleus, whereas the *Tfeb*^*S141D*^ mutant exhibited significantly increased cytosolic localization compared with the control (Fig. S1b).

**Figure 7:**
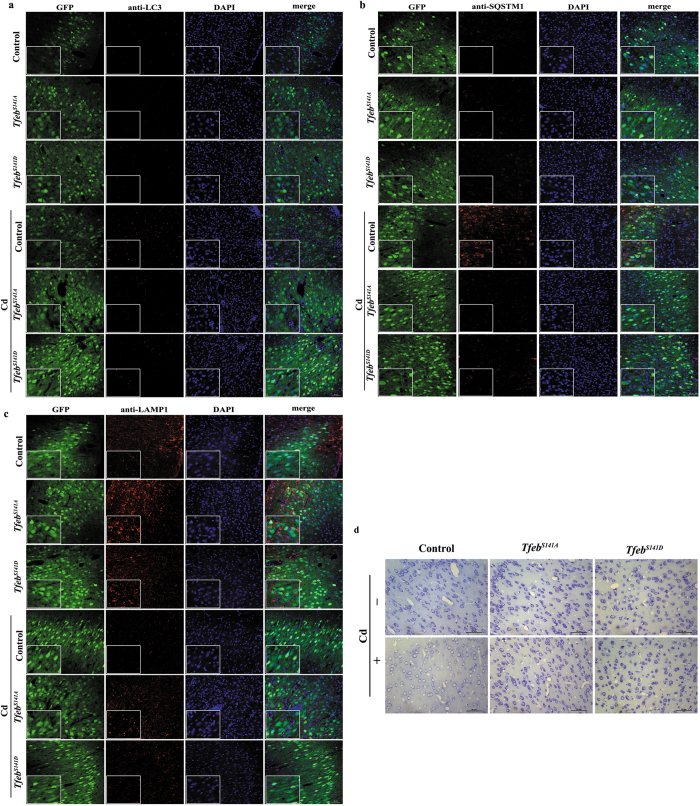
TFEB is a new therapeutic target for Cd-induced neurotoxicity *in vivo.* *Tfeb* was overexpressed by stereotaxic delivery of AAV before Cd exposure. Immunofluorescence analysis of (**a**) LC3B expression, (**b**) SQSTM1 expression, (**c**) LAMP1 expression and (**d**) Nissl staining in brain tissue. Panels on the left are high magnification images. The results are expressed as fold changes compared with the control. The values are presented as the means ± SEM. **p < 0.01 versus the control group, #p < 0.05, ^##^p < 0.01 versus the Cd group. (n = 12~15).

To determine whether *Tfeb* overexpression can restore autophagic flux and lysosomal function, immunofluorescence staining of cortical sections was used. Immunofluorescence analysis of the Cd-treated cortical neurons revealed inhibition of autophagy as evidenced by the marked accumulation of LC3II and p62, this accumulation was attenuated by *Tfeb* overexpression regardless of the subcellular location of TFEB (Fig. 7a,b and S3f). Accordingly, LAMP1 levels were dramatically decreased in Cd-treated cortical neurons (Fig. 7c and S3f), a finding that is consistent with a dramatic impairment of lysosomal function. In addition, a concomitant increase in the LAMP1 protein levels was observed upon *Tfeb* overexpression, with no significant difference between *Tfeb*^*S141A*^ and *Tfeb*^*S141D*^ (Fig. 7c and S3f). These results suggested the restoration of autophagic flux and lysosomal function by *Tfeb* overexpression regardless of the subcellular location of TFEB.

Then, we investigated whether the restoration of autophagic flux and lysosomal function by *Tfeb* overexpression could protect neurons from Cd-induced cell death. As shown in Fig. 7d, Cd treatment resulted in a loss of Nissl bodies, as visualized with Nissl staining. Nissl staining in the Ml area was reduced, but not lost, indicating the initiation of cell damage. Consistent with the restoration of autophagic flux and lysosomal function, *Tfeb*^*S141A*^ and *Tfeb*^*S141D*^ overexpression resulted in a significant increase in Nissl staining (Fig. 7d), identifying a protective role of TFEB in Cd neurotoxicity *in vivo*.

## Discussion

Cd-induced neurotoxicity includes cell death, neurobiochemical changes (neurotransmitters and receptors), and physiological/behavioural changes^25^,^26^. However, its underlying mechanism remains unclear. In our study, we first demonstrate that **(i)** Cd blocks autophagic flux via the inhibition of autophagosome-lysosome fusion and the disruption of lysosomal function; **(ii)** Cd decreases TFEB expression but induces nuclear translocation of endogenous TFEB and TFEB-target gene expression, associated with compromised lysosomal function or a compensatory effect after the impairment of the autophagic flux; **(iii)** TFEB plays a critical role in enhancing autophagic flux and cytoprotection against Cd injury, and this protective effect is independent, at least in part, of its role as a nuclear transcription factor. The current study provides new evidence that the stimulation of TFEB-dependent autophagy-lysosome machinery protects against Cd-induced neurotoxicity *in vitro* and *in vivo.*

Autophagy is a highly conserved cellular degradation process through which cells remove damaged organelles and toxic macromolecules. Although under certain circumstances pathologically increased autophagy has been implicated in cell death^27^, under most circumstances autophagy is considered a cytoprotective mechanism. Basal levels of autophagy are important for maintaining cellular homeostasis and appear to be essential for normal cellular function and the survival of terminally differentiated cells, such as neurons^28^,^29^. Recently, various studies have demonstrated that dysfunction of autophagy is implicated in neuronal cell loss in neurodegenerative diseases. For example, defects in autophagic flux lead to aggregate-prone proteins such as huntingtin and a-synuclein, which are accumulated in Huntington’s and Parkinson’s diseases^30^,^31^. The role of autophagy in Cd toxicity is still a controversial topic. Various studies have demonstrated that Cd exposure induced a significant degree of autophagy, which led to cell death in the liver^32^, osteoblasts^33^, and mesenchymal stem cells^34^. In contrast, impaired autophagy plays a vital role during Cd-induced neurotoxicity^35^ and nephrotoxicity^36^. Consistent with previous studies, we show that Cd inhibits autophagic flux, which subsequently may have an impact on Neuro-2a cell death. Thus, our data reveal a potential common mechanism resulting from neuronal cell death due to chronic (neurodegenerative diseases) and acute (Cd) insults.

In the early 1950 s, Christian De Duve identified a new cellular structure, the lysosome, which was defined as the cell’s “suicide bag”^37^. The lysosome consists of more than 50 acid hydrolases that exert a digestive function and 120 membrane proteins that maintain lysosomal integrity and regulating lysosomal trafficking, fusion, and intra-lysosomal pH, which are critical for eliminating cellular debris, damaged organelles, and invading microorganisms^38^,^39^,^40^. Autophagy targets almost all organelles including mitochondria, ER, peroxisomes, and ribosomes, but all of these cargos are delivered to one organelle, the lysosome, for degradation. Lysosomes are, therefore, essential for autophagy, and lysosomal dysfunction should result in the perturbation of autophagy. The results of the present study showed that Cd impaired lysosomal function by reducing the levels of lysosomal-associated membrane proteins, inhibiting lysosomal proteolysis and altering lysosomal pH. Targeting the lysosome as a Cd-triggered neurotoxicity therapy may be important and beneficial. LAMP1 is the predominant lysosomal membrane protein and function, in part, to maintain the integrity of the lysosomal membrane in various cells^41^. LAMP1 knockdown is associated with decreased lysosome integrity, increased ROS production, and decreased cell viability^42^. Moreover, LAMP-1 is also a biomarker of lysosomal biogenesis^43^. Cd induced a decrease in LAMP1 levels, suggesting that Cd decreases the amount or size of lysosomes and disrupts lysosomal biogenesis.An exciting and promising aspect of our study is the ability of the transcription factor TFEB to enhance autophagy and lysosomal function and subsequently rescue Cd-triggered neurotoxicity. TFEB, a member of the MiT/TFE helix-loop-helix subfamily, initiates a lysosomal biogenesis program, thus stimulating the overall degradation capacity of cells^44^. Our study observed a decrease in the level of TFEB, and predominant nuclear localization of TFEB was detected in Cd-treated Neuro-2a cells. Consistently, TFEB mobilizes into the nucleus when lysosomal function is compromised or as a compensatory response to autophagy inhibition^23^,^45^, suggesting that TFEB is activated in response to lysosomal stress induced by Cd and autophagic flux impaired by Cd. Moreover, emerging studies have demonstrated that TFEB modulates autophagy by positively regulating autophagosome formation and autophagosome–lysosome fusion both *in vitro* and *in vivo*^46^. TFEB overexpression effectively reduces amyloid β accumulation in neurons^47^, and rescues midbrain dopamine neurons from α-synuclein toxicity^48^. Certain chemical compounds increase TFEB expression and provide therapeutic efficacy in other lysosomal storage diseases. Some of these compounds have already been used in clinical trials. For example, β-cyclodextrin and fisetin activate TFEB and improve autophagy-lysosomal machinery^49^,^50^. Our study observed that *Tfeb* overexpression efficiently protected against the inhibition of autophagosome-lysosome fusion, the accumulation of an autophagic substrate, and the reduction of lysosomal function in Cd-induced neurotoxicity. Importantly, our study showed that restoration of lysosomal function and autophagic flux by *Tfeb* overexpression is accompanied by improved neuronal survival, hinting at a prominent role of TFEB-mediated replenishment of the autophagy-lysosome machinery in alleviating Cd-triggered neurotoxicity.

As a nuclear transcription factors, TFEB regulates both autophagy and lysosomal function via rapid translocation to the nucleus from the cytosol and lysosomes. However, our results demonstrated that TFEB with an inactivated mutant nuclear localization signal (*Tfeb*^*S141D*^) restores Cd-induced impairment of autophagic flux and lysosomal function, suggesting that the cytosolic localization of TFEB also plays an important role in lysosomal function and autophagy. To the best of our knowledge, this report is the first to demonstrate that translocation of TFEB to the nucleus may not be exclusively required for enhancing lysosomal function and autophagic flux in Cd-induced neurotoxicity. Moreover, the *Tfeb*^*S141A*^ mutant exhibited significantly increased TFEB target-genes expression compared with control plasmids; conversely, the phosphomimetic mutant *Tfeb*^*S141D*^ was unable to enhance TFEB target-gene expression (Fig. 6e). Unlike the previous study^51^, we can exclude the possibility that exogenous *Tfeb*^*S141D*^ induces endogenous nuclear TFEB expression to create a positive feedback loop in Cd-induced neurotoxicity. Further investigation is required to determine how cytosolic TFEB functions in autophagic flux in our model.

Cd can access the central nervous system directly depending on blood-brain barrier vulnerability or bypass the blood-brain barrier through olfactory pathways, producing neurotoxic effects^52^,^53^. Consistent with experimental studies in the CNS of rats and rabbits exposed to Cd, our study showed extensive damage in the cerebral cortices (Fig. S2)^54^,^55^,^56^. Genetic activation of TFEB by AAV-TFEB, the master transcriptional activator of the autophagy-lysosomal system, is shown to be beneficial in rescuing Pompe disease mouse model, enhancing autophagy and lysosome biogenesis in the liver, and reducing accumulation of Alpha-1-anti-trypsin in the livers of PiZ mice^57^,^58^. Here, we chose the M1 area to investigate whether *Tfeb* overexpression protects from Cd injury by stereotaxic delivery of AAV (Fig. S4). Consistent with the data obtained *in vitro*, both nuclear and cytosolic *Tfeb* overexpression in the M1 area promotes neuron survival by enhancing autophagic flux and lysosomal function, suggesting that *Tfeb* gene transfer may serve as a novel strategy for intervening Cd neurotoxicity. However, the cytoprotective role of *Tfeb* gene transfer by stereotaxic delivery was confined to the area where the virus was injected. The problems of the current system are expected to be overcome by further improvements, including using *Tfeb* transgenic mice and modifying the vector or virus delivery method such as tail vein injections of virus so that more neurons can be transfected.

Collectively, these findings emphasize the importance of the autophagic –lysosomal pathway in Cd-induced neurotoxicity and that therapeutic strategies aimed at rescuing and/or enhancing the TFEB-dependent pathway may have a broad impact on human health.

## Materials and Methods

### Cell culture and treatment

Mouse neuroblastoma cells (Neuro-2a cells) were obtained from the Cell Bank of the Institute of Biochemistry and Cell Biology (Shanghai, China) and grown in DMEM culture medium (HyClone, SH30022.01B) plus 10% fetal bovine serum (HyClone, SV30087.02) and 100 units/ml of penicillin/streptomycin (Sigma, P4333) at 37 °C in a humidified atmosphere containing 5% CO_2_. For the cadmium chloride (CdCl_2_, Sigma, 439800) treatments, the cells were grown to 80% confluence and treated with different concentrations (0, 12.5, 25, or 50 μM) for 24 h. For treatment with chloroquine (CQ) (Sigma, C6628), CQ was dissolved in distilled deionized water to produce a stock solution, which was then appropriately diluted with cell culture medium before application.

### Cell viability assay

In accordance with the manufacturer’s instructions, the Cell Counting Kit-8 (CCK-8) (Dojindo, CK04) assay was used to test cell viability. Briefly, 1 × 10^4^ cells were seeded in 96-well plates. Then, 90 μl of medium and 10 μl of CCK-8 reagents were added to a subset of wells under different treatments and incubated for 1 h at 37 °C, Then, the OD value was measured at 450 nm using an Infinite™ M200 Microplate Reader (Tecan, Mannedorf, Switzerland).

### Transfection

According to the manufacturer’s instructions, cells were transfected with Opti-MEM^®^ I reduced serum media and Lipofectamine 2000 (Invitrogen, 11668–019). For *Tfeb* overexpression, two plasmids, pEGFP-N1-*Tfeb*^*S141A*^, pEGFP-N1-*Tfeb*^*S141D*^, and control plasmids designed by Invitrogen Corporation (Shanghai, China) as well as GFP-LC3 plasmid (Cell Biolabs, CBA-401) were transfected into Neuro-2a cells. At 24 h after transfection, the cells were exposed to 50 μM Cd for 24 h.

### RFP-GFP-LC3 assay

To evaluate the numbers of autophagosomes and autolysosomes and to analyse autophagic flux^59^, Neuro-2a cells were infected with lentiviruses carrying expression cassettes that encode tandem fluorescence-tagged LC3B. Briefly, Neuro-2a cells were grown on coverslips at 2 × 10^5^ cells/well. Then, the cells were infected using a Premo™ Autophagy Tandem Sensor RFP-GFP-LC3B (HANBIO, China), in which GFP is more sensitive to acidic conditions than RFP. Twenty-four hours after infection, the Neuro-2a cells were treated with 50 μM Cd for 24 h. All the samples were examined under a Zeiss confocal laser scanning microscope (Zeiss, LSM 780) equipped with a 63x oil immersion objective.

### DQ-BSA lysosomal activity assay

Cells (1 × 10^4^) were first loaded with 10 μg/ml DQ™ Red BSA (Life Technologies, D-12051) at 37 °C for 6 h prior to treatment with 50 μM Cd for various times. Following treatment, the cells were washed with PBS to remove excessive DQ-BSA and lysed in 1% Triton X-100 in 50 mM Tris-HCL (pH 8.8) solution. The fluorescence intensity of the lysates was quantified using a Tecan Infinite M200 Pro plate reader (excitation: 590 and emission: 620)^60^.

### CTSD activity assay

The catalytic activity of CTSD was determined using CTSD activity fluorometric assay kits (BioVision, K143–100). Briefly, 1 × 10^6^ cells were collected by centrifugation and lysed in 200 μl of chilled cell lysis buffer. Then, 50 μl of cell lysate was transferred into 96-well plates and mixed with the reaction. The samples were read in a fluorometer with 328 nm excitation and 460 nm emission filters. The activity was normalized to the samples’ protein concentration.

### Lysosomal pH measurement

The quantification of the lysosomal pH was performed using LysoSensor Green DND-189 (Invitrogen, L7535)^61^. Briefly, the cells were loaded with 1 μ M LysoSensor Green DND-189 in prewarmed regular medium for 5 min at 37 °C. Then, the cells were washed twice with PBS. Following treatment, the fluorescence intensity of the cells was quantified using an Infinite™ M200 Microplate Reader (excitation: 485 and emission: 530).

### Immunocytochemical analysis of Neuro-2a cells

Immunofluorescence was performed according to standard procedures. In brief, cells were grown on gelatine-coated glass coverslips. After the cells were incubated with the indicatedagent, they were fixed with 4% (w/v) paraformaldehyde in PBS for 30 min followed by permeabilization with 0.25% Triton X-100 in PBS for 10 min at room temperature. Then, the cells were blocked with 10% BSA in PBS. The fixed cells were incubated with rabbit anti-TFEB (1:500, Bethyl Laboratories, A303–673A), rabbit anti-LC3 (1:250, Sigma, L7543), rabbit anti-LAMP-1 (1:100, Abcam, ab24170), or rat anti-LAMP-1 (1:50, Santa Cruz Biotechnology, sc-19992) antibody in immuno staining dilution buffer at 4 °C overnight. The slides were then washed five times with PBS and incubated with an Alexa Fluor^®^ 568 donkey anti-rabbit IgG (H+L) antibody (Life Technologies, A10042), an Alexa Fluor^®^ 647 donkey anti-rabbit IgG (H+L) antibody (Life Technologies, A-31573) or an Alexa Fluor^®^ 568 goat anti-rat IgG (H+L) antibody (Life Technologies, A-11077) at a 1:200 dilution for 1 h at 37 °C. DAPI Staining Solution (Beyotime, C1005) was used for nuclear counterstaining. The coverslips were mounted on glass slides using Antifade Mounting Medium (Beyotime, P0126). The stained samples were examined using a Zeiss confocal laser scanning microscope (Zeiss, LSM780) equipped with a 63x or 40x oil objective. The colocalization coefficient was calculated using Zeiss LSM 780 software between stacks of images from 2 channels. At least 30 cells were counted for each experiment.

### Western blot analyses

Cells were harvested and washed with cold phosphate-buffered saline (PBS). The proteins were extracted with RIPA Cell Lysis Buffer (Beyotime Institute of Biotechnology, Haimen, China) and maintained on ice for at least 30 min. The lysates were centrifuged at 12,000 g at 4 °C for 10 min, and the supernatant was transferred to a fresh tube. After the protein concentration was measured using the bicinchoninic acid (BCA) method, an equal quantity of total protein per lane was separated by sodium dodecyl sulfate-polyacrylamide gel electrophoresis (SDS-PAGE) and transferred to polyvinylidene fluoride (PVDF) membranes. The membranes were blocked with 10% non-fat dry milk powder in PBS for 1 h at room temperature and then incubated overnight at 4 °C with specific antibodies against LC3 (1:1000, Sigma, L7543), SQSTM1/p62 (1:1000, Abcam, ab56416), TFEB (1:2000, Bethyl Laboratories, A303–673A), LAMP-1(1:1000, Abcam, ab24170), and ACTB (1:5000, Sigma, A5441). After the membranes were incubated with the primary antibody, they were washed three times in TBST and then incubated with corresponding HRP-conjugated anti-mouse or anti-rabbit (Beyotime, A0208 or A0216) secondary antibody for 1 h at room temperature. Next, the membranes were washed and visualized using a Luminata Forte Western HRP Substrate (Merck Millipore, WBLUF0500). The bands were quantified with Image J software.

### Real-time RT-PCR analysis

RNA was isolated from Neuro-2a cells treated or untreated with Cd. cDNA synthesis was performed using the AffinityScript QPCR cDNA Synthesis Kit (Stratagene, Agilent Technologies) with 1 mg of total RNA and random primers. The resulting RT product was expanded using the NovoStart^®^ SYBR qPCR Supermix (E090-01A, novoprotein) and specific primers for *Tfeb* (forward: AAGGTTCGGGAGTATCTGTCTG; reverse: GGGTTGGAGCTGATATGTAGCA); Gba (forward: GCCAGGCTCATCGGATTCTTC; reverse: GAGTGCTCTCGTAAC GGCT). Atb6v1g1 (forward: CCCAGGCTGAAATTGAACAGT; reverse: TTCTGG AGGACGGTCATCTTC).*Clcn7* (forward: CGCCAGTCTCATTCTGCACT; reverse: GAGGATCGACTTCCGGGTC). Detection was per-formed with the Mx3000P QPCR system (Stratagene, Agilent Technologies). Threshold cycle (Ct) values were obtained for each gene at the different time points using instrument software. Differences in the levels of gene expression over time were determined for each condition by relative quantification using the Delta Ct method.

### Animal surgical procedures and treatment

Five-week-old male C57Bl/6 mice were purchased from the Experimental Animal Center of the Third Military Medical University (Chongqing, China) and housed two to three per cage with ad libitum access to food and water during a 12-h light/dark cycle. Three AAVs, AAV-SYN-EGFP, AAV- *Tfeb*^*S141A*^-SYN-EGFP and AAV-*Tfeb*^*S141D*^-SYN-EGFP, were all constructed and packaged by OBio company (Shanghai, China); synapsin I (SYN) is a type of neuron-specific promoter. For stereotaxic delivery of the virus^62^, the mice were subject to general anaesthesia using pentobarbital sodium (1 g/kg), which was injected intraperitoneally. Then, the mice were placed in a stereotaxic frame and injected with 0.2~0.4 μl of purified and concentrated AAV unilaterally into the left cerebral cortex (AP = 1.1 mm, ML = −1.5 mm, DV = 0.3 mm) using glass microelectrodes at a slow rate (~50–100 nl/min). The injection microelectrode was slowly withdrawn 5 min after virus infusion. Cd treatment was initiated 3 weeks after AAV vector injection. Mice received 1 intraperitoneal injection of Cd (2 mg/kg/day) dissolved in 0.9% physiological saline, per day for 7 consecutive days. Control mice received saline injections only. An average of 12 to 15 mice was used in each group. All animal experimental procedures were conducted in accordance with institutional animal welfare guidelines and were approved by the Third Military Medical University Animal Care and Use Committee.

### Nissl staining and immunofluorescence in mouse brain tissue

After the mice were treated, they were perfused transcardially with 0.9% saline followed by 4% paraformaldehyde (PFA) in PBS under pentobarbital anaesthesia. Then, the brains were removed, fixed overnight in 4% PFA, and cryoprotected in 30% sucrose. For Nissl staining, we used paraffin-embedded brain sections. For immunofluorescence and fluorescent Nissl staining, mouse brains embedded in OCT were used. Paraffin-embedded coronal sections were cut into 10-μm thick sections for Nissl staining. These sections were processed with 1.0% cresyl violet, dehydrated, and cover slipped with Entellan. For immunofluorescence, mouse brains were sectioned at a thickness of 30 μm. The sections were incubated in 0.3% H_2_O_2_ in PBS for 10 min, then permeabilized for 30 min using a 1% Triton X-100/PBS solution, and blocked in a solution of 10% normal donkey serum and 0.3% Triton X-100 in PBS for 2 h at room temperature. The sections were incubated in primary antibody solutions overnight at 4 °C. The primary antibodies used included rabbit anti-TFEB (1:500, Bethyl Laboratories, A303–673A), rabbit anti-LC3 (1:200, Abcam, ab64781), rabbit anti-LAMP-1 (1:100, Abcam, ab24170) or rat anti-LAMP-1 (1:50, Santa Cruz Biotechnology, sc-19992). The sections were further incubated with appropriate Alexa Fluor 568- or 647-conjugated secondary antibodies for 2 h at room temperature. Cell nuclei were visualized by staining with DAPI Staining Solution (Beyotime, C1005) for 10 min at room temperature. Then, the sections were mounted on glass slides, and digital images were acquired at 40x or 200x magnification using a confocal laser scanning microscope. Fluorescence intensity was quantified with Zeiss LSM 780 software.

### Statistical analysis

The data are expressed as the mean ± SEM. The data for the groups were compared using one-way ANOVA (Bonferroni’s multiple comparison test) for parametric (normality and equal variance passed) data. The Kruskal-Wallis ANOVA-based test on ranks followed by Dunn’s post hoc test was used for nonparametric data (i.e., those that failed normality and/or equal variance tests). For experiments with only 2 groups, the 2-tailed Mann-Whitney rank-sum test (nonparametric) or the 2-tailed unpaired Student t-test was performed. In all analyses, the null hypothesis was rejected at the 0.05 level. All of the counting analyses were performed in a blinded manner.

## Additional Information

**How to cite this article:** Pi, H. *et al*. Enhancing lysosomal biogenesis and autophagic flux by activating the transcription factor EB protects against cadmium-induced neurotoxicity. *Sci. Rep.* **7**, 43466; doi: 10.1038/srep43466 (2017).

**Publisher's note:** Springer Nature remains neutral with regard to jurisdictional claims in published maps and institutional affiliations.

## Supplementary Material

Supplementary Information
