# Supplementary material for: Enhancing lysosomal biogenesis and autophagic flux by activating the transcription factor EB protects against cadmium-induced neurotoxicity
Source: Sci Rep. 2017 Feb 27;7:43466. doi: 10.1038/srep43466 (PMC5327481; doi:10.1038/srep43466)
Supplement: Supplementary Information [file srep43466-s1.pdf]

Supplemental Material to:

Enhancing lysosomal biogenesis and autophagic flux by activating the transcription factor EB protects against cadmium-induced neurotoxicity

Huifeng Pi, Min Li, Li Tian, Zhiqi Yang, Zhengping Yu, Zhou Zhou

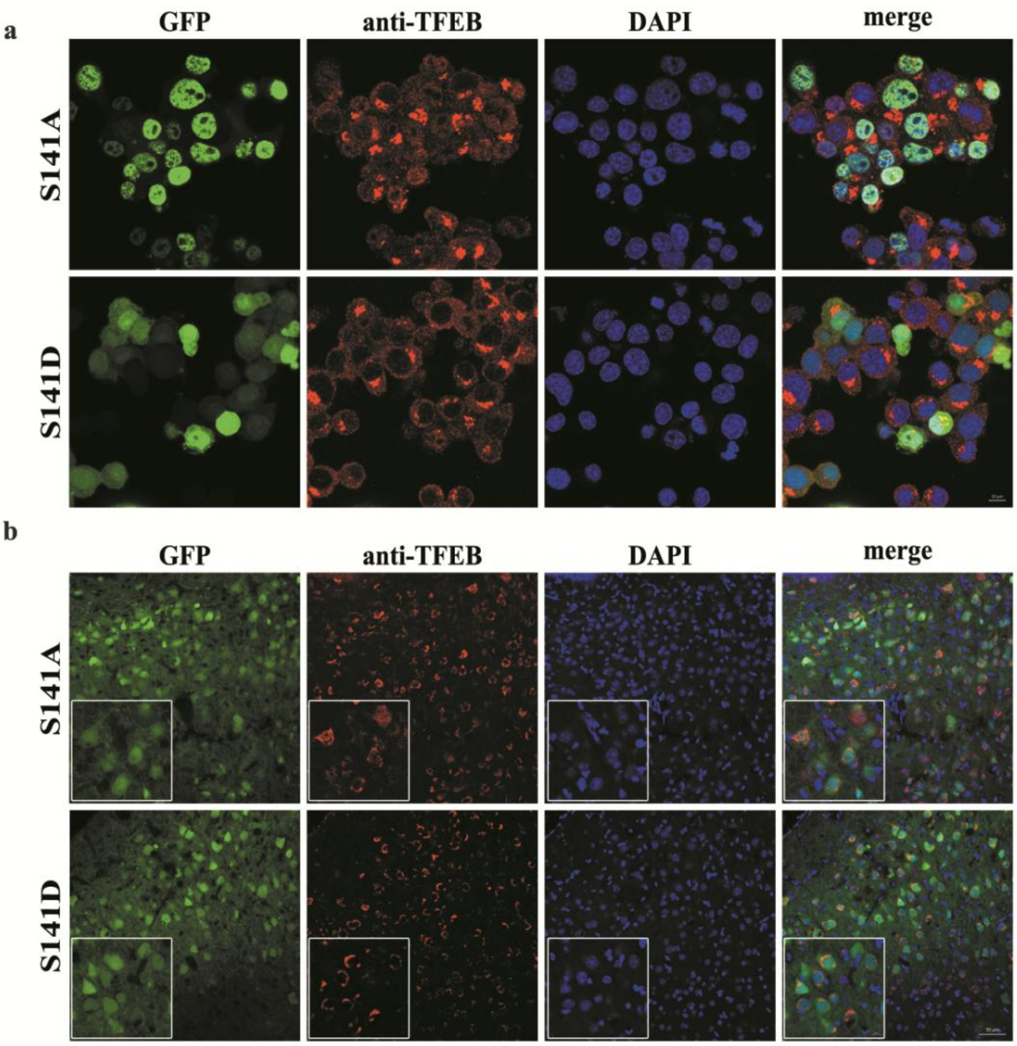

Figure S1. The *Tfeb*^{S141A} mutant nearly translocates into nucleus; conversely, the mutant *Tfeb*^{S141D} is unable to translocate into the nucleus. Immunofluorescence of **A**. Neuro-2a cells and **B**. brain tissue with anti-TFEB antibody and DAPI. Panels on the left are high magnifications.

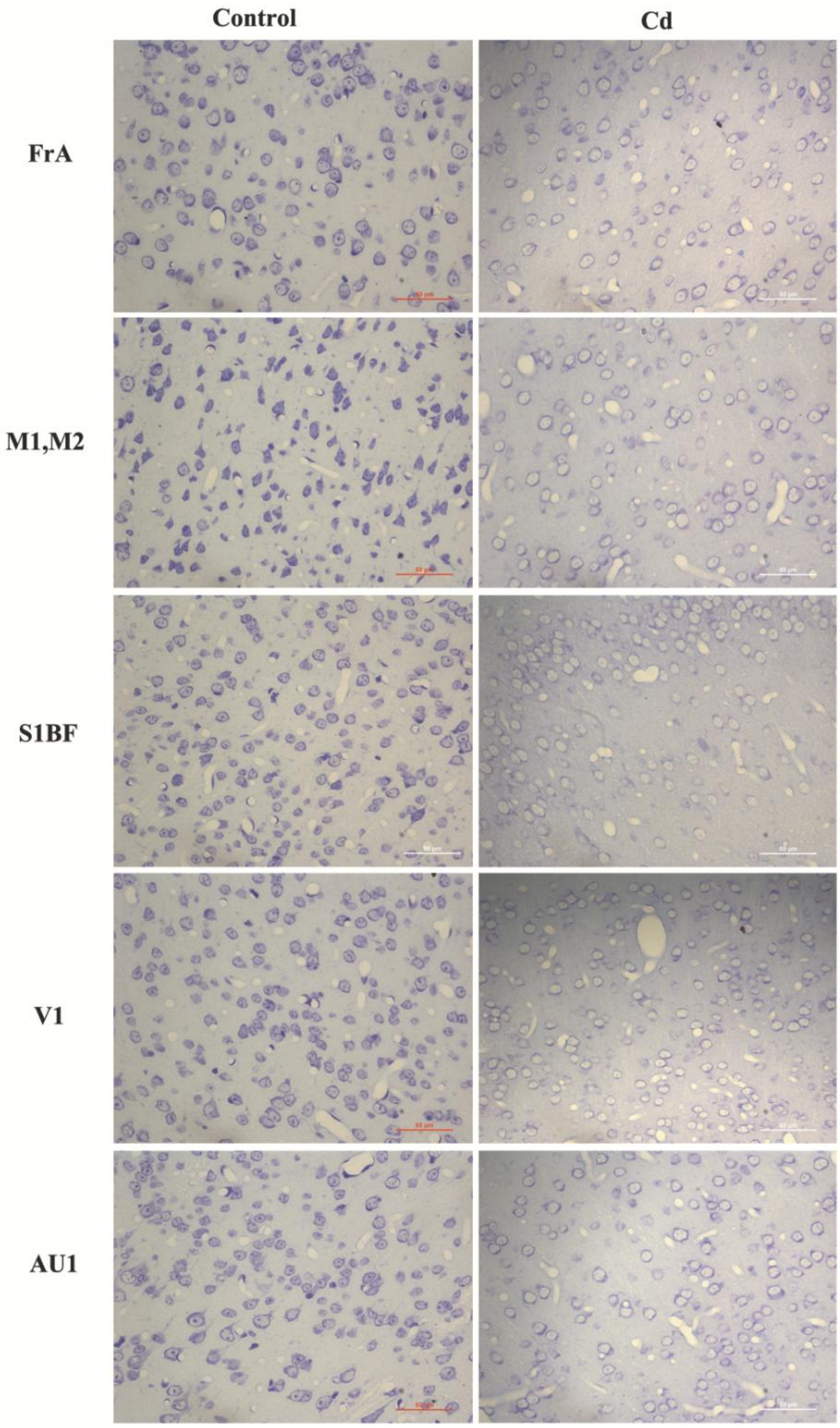

Figure S2. Cd exposure leads to extensive damage in the cerebral cortices of mice. Nissl staining of the brain tissue.

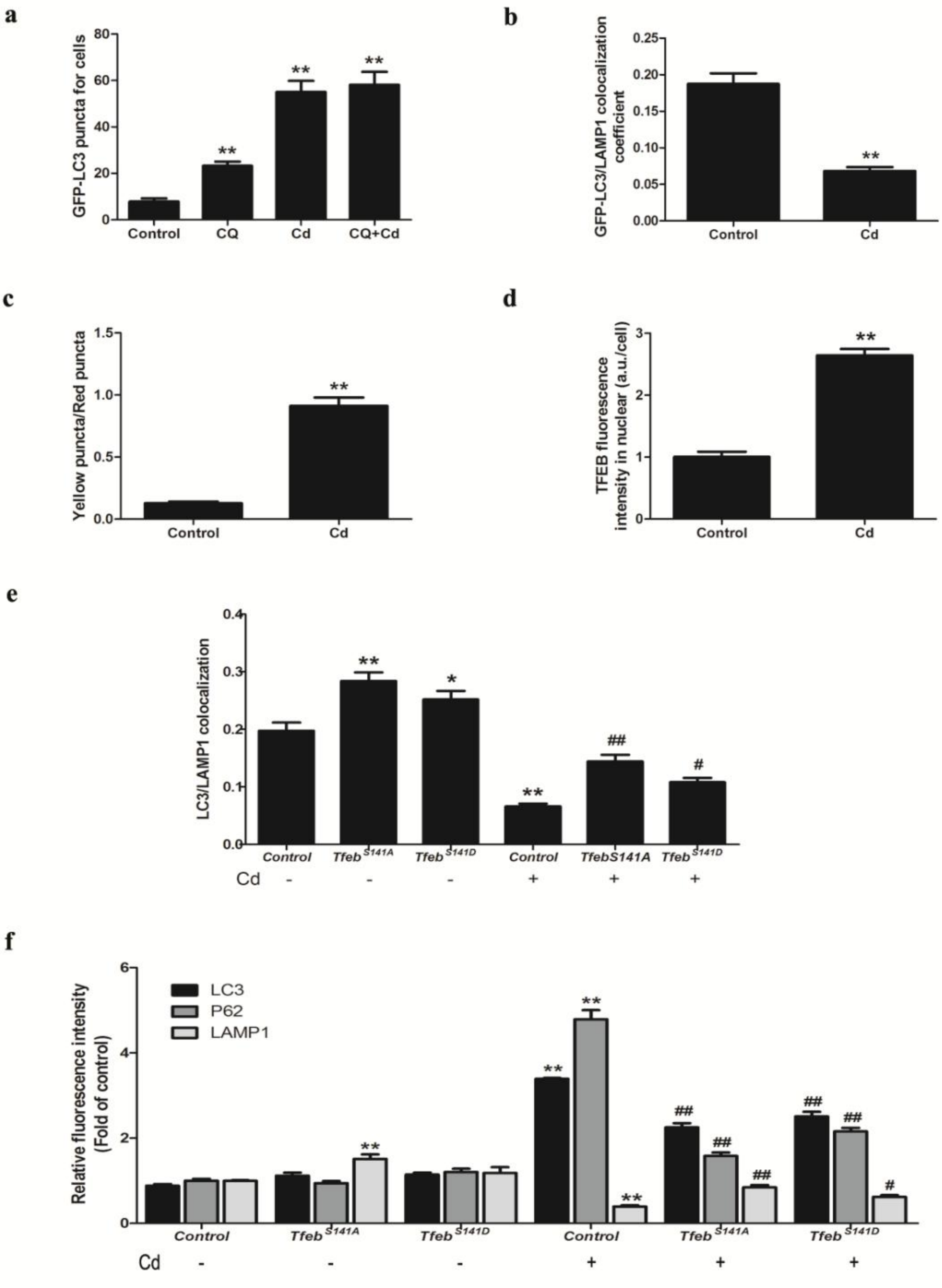

Figure S3. Quantification of immunocytochemical analysis of Neuro-2a cells and in mouse brain tissue. (**a**) Quantification of Fig. 2c. (**b**) Quantification of Fig. 3a. (**c**) Quantification of Fig. 3b. (**d**) Quantification of Fig. 5c. (**e**) Quantification of Fig. 6b. (**f**) Quantification of Fig. 7a-c.
